# m^6^A methyltransferase METTL3 promotes retinoblastoma progression via PI3K/AKT/mTOR pathway

**DOI:** 10.1111/jcmm.15736

**Published:** 2020-10-08

**Authors:** Han Zhang, Ping Zhang, Chongde Long, Xinqi Ma, Hao Huang, Xielan Kuang, Han Du, Han Tang, Xiangtian Ling, Jie Ning, Huijun Liu, Xizhi Deng, Yuxiu Zou, Renchun Wang, Hao Cheng, Shuibin Lin, Qingjiong Zhang, Jianhua Yan, Huangxuan Shen

**Affiliations:** ^1^ State Key Laboratory of Ophthalmology Zhongshan Ophthalmic Center Sun Yat‐sen University Guangzhou China; ^2^ Biobank of Eye State Key Laboratory of Ophthalmology Zhongshan Ophthalmic Center Sun Yat‐sen University Guangzhou China; ^3^ The Second Clinical Medicine School of Lanzhou University Lanzhou China; ^4^ Department of Ophthalmology The First Affiliated Hospital of Guangzhou Medical University Guangzhou China; ^5^ Center for Translational Medicine The First Affiliated Hospital Sun Yat‐sen University Guangzhou China

**Keywords:** apoptosis, METTL3, PI3K/AKT/mTOR, proliferation, retinoblastoma

## Abstract

Retinoblastoma (RB) is a common intraocular malignancy in children. Due to the poor prognosis of RB, it is crucial to search for efficient diagnostic and therapeutic strategies. Studies have shown that methyltransferase‐like 3 (METTL3), a major RNA N (6)‐adenosine methyltransferase, is closely related to the initiation and development of cancers. Nevertheless, whether METTL3 is associated with RB remains unexplored. Therefore, we investigated the function and mechanisms of METTL3 in the regulation of RB progression. We manipulated METTL3 expression in RB cells. Then, cell proliferation, apoptosis, migration and invasion were analysed. We also analysed the expression of PI3K/AKT/mTOR pathway members. Finally, we incorporated subcutaneous xenograft mouse models into our studies. The results showed that METTL3 is highly expressed in RB patients and RB cells. We found that METTL3 knockdown decreases cell proliferation, migration and invasion of RB cells, while METTL3 overexpression promotes RB progression in vitro and in vivo. Moreover, two downstream members of the PI3K/AKT/mTOR pathway, P70S6K and 4EBP1, were affected by METTL3. Our study revealed that METTL3 promotes the progression of RB through PI3K/AKT/mTOR pathways in vitro and in vivo. Targeting the METTL3/PI3K/AKT/mTOR signalling axis could be a promising therapeutic strategy for the treatment of RB.

## 1 INTRODUCTION

Retinoblastoma (RB) is a highly aggressive paediatric ophthalmological malignancy that commonly affects the eyes of children under 5 years old and is responsible for 5% of blindness in children.^1^, ^2^ Retinoblastoma initiation occurs with exceptionally high efficiency in response to the loss of functional pRB protein, which is encoded by the RB1 gene.^3^ Retinoblastoma is derived from the immature cells of the retina and often extends along the optic nerve into the brain or metastasizes distally to other organs.^4^ Retinoblastoma can lead to devastating consequences, including blindness and even death.^5^ Recently, Yang et al found that self‐generated oxygen could mediate the therapy of tumours through scavenging in hypoxic conditions.^6^ Studies show that establishment of hypoxia has been identified as a significant step in RB tumour progression.^7^, ^8^, ^9^ Currently, although it is widely accepted that mutations in the 2 alleles of the RB tumour suppressor gene RB1 are triggers for this cancer,^10^ the underlying mechanisms of RB progression are not fully understood.

Recent studies have uncovered the critical functions of N6‐methyladenosine (m^6^A), an epigenetic modification of RNA, in the regulation of cancer initiation and progression. N6‐methyladenosine is the most pervasive internal post‐transcriptional modification within eukaryotic messenger RNAs (mRNAs).^11^ Modification of mRNA with m^6^A occurs cotranscriptionally via a complex composed of multiple subunits, including the catalytic enzyme methyltransferase‐like 3 (METTL3), methyltransferase‐like 14 and Wilms tumour 1‐associated protein.^12^, ^13^ Emerging evidence suggests that METTL3 has diverse functions in different cancers. METTL3 regulates oncogene expression by affecting mRNA processing, stability and translation, and it facilitates the progression of different types of cancers, including lung cancer,^14^ breast cancer,^15^ colorectal carcinoma,^16^ bladder cancer ^17^ and hepatoblastoma.^18^ In addition, METTL3‐depleted pancreatic cancer cells showed higher sensitivity to anticancer reagents such as gemcitabine, 5‐fluorouracil, cisplatin and irradiation, suggesting that METTL3 is a potent target for enhancing therapeutic efficacy.^19^ However, the function of METTL3 in RB and the mechanism of METTL3 in the progression of RB remain unknown.

In this study, we investigated the function and mechanism of METTL3 in the pathogenesis of RB and revealed that METTL3 promotes RB progression in vitro and in vivo, suggesting that METTL3 may be a novel therapeutic target for RB treatment.

## 2 MATERIAL AND METHODS

### 2.1 Clinical tissue samples and cell lines

Retinoblastoma tissues in paraffin were obtained from Zhongshan Ophthalmic Center, Sun Yat‐sen University. We obtained approval for this study from the Zhongshan Ophthalmic Center Ethics Committee. The human RB cell lines Y79 and WERI‐Rb‐1 were purchased from American Type Culture Collection. All cell lines were cultured in RPMI 1640 basic (1×) growth medium (Gibco; Thermo Fisher Scientific, USA, New York) containing 10% FBS (Fatal Bovine Serun) (Gibco; Thermo Fisher Scientific, USA, New York) and 1% penicillin and streptomycin (HyClone; GE Healthcare Life Science, USA, New York), and they were maintained at 37°C in a 5% CO_2_ atmosphere.

### 2.2 Plasmids and transfection

For METTL3 knockdown, Y79 and WERI‐Rb‐1 cells were transfected with pLKO.1‐TRC negative control vectors and METTL3 knockdown plasmids using Lipofectamine™ 3000 Transfection Reagent (Invitrogen; Thermo Fisher Scientific, USA, New York) following the manufacturer's instructions. The lentivirus constructs were generated to up‐regulate METTL3. Y79 and WERI‐Rb‐1 cells were stably transfected with pCDH‐Vec negative control vectors and METTL3 up‐regulated lentivirus. The plasmids were gifts from Professor Shuibin Lin.^14^ Lentiviral transfection was performed as described previously.^20^ Briefly, cells (2 × 10^5^/mL) were seeded in six‐well plates in 2 mL of culture media and then were infected with the lentiviruses. Polybrene (10 μg/mL; Sigma, USA, New York) was added to the lentiviruses to enhance infection efficiency. Pooled stable populations of Y79 and WERI‐Rb‐1 cells were generated by treatment with puromycin (20 mg/mL; Solarbio Life Science, China, Beijing) for 1 week.

### 2.3 Cell Counting Kit‐8 assays

For the CCK‐8 (Cell Counting Kit‐8) assays, 5000 cells/well were seeded in 96‐well plates and cultured for 0, 24 and 48 hours. 10 μL of CCK‐8 (Dojindo, Japan, Tokyo) solution was added to the cells for a 4 hours incubation at 37°C in 5% CO_2_ atmosphere, and then, the absorbance at 450 nm was detected by a microplate reader (BioTek Instruments, Winooski, VT).

### 2.4 Apoptosis

Cells (1 × 10^6^) were seeded in six‐well plates, and after being cultured for 24 hours, they were collected and centrifuged. 5 μL of PI(Propidium Iodide) 5 μL of FITC(Fluorescein Isothiocyanate) (BD Pharmingen, USA, New York) were added after the cells were pelleted and resuspended in 100 μL of 1× binding buffer. Apoptosis was detected by flow cytometry (LSRFortessa, BD, USA, New York) after 15 minutes of incubation in the dark at room temperature. The results were analysed by FlowJo 7.6.2.

### 2.5 Transwell assays

We used a Corning™ 8 μm pore polycarbonate membrane 24‐well transwell chamber. A 200 μL suspension (FBS‐free 1640 media) of Y79 cells or WERI‐Rb‐1 cells was seeded in each upper chamber at 40 000 cells/mL, and the migration capacity of the cells was detected 24 hours after plating; the lower chamber contained 20% FBS 1640 media. Finally, the migrating cells were fixed with methanol (Boster Biological Technology, China, Beijing) for 15 minutes at room temperature. After washing with 1× PBS (Gibco; Thermo Fisher Scientific, USA, New York), the upper chamber was stained with crystal violet (Solarbio Life Science, China, Beijing) for 15 minutes at room temperature. Compared to the transwell migration assay, the difference in the invasion assay was that the bottom of the upper chamber was pre‐coated with Matrigel (BD Biosciences, Bedford, MA, USA), and the number of cells was 20 000 cells/mL, and they were in FBS‐free 1640 media. Five fields of 8 μm pore polycarbonate membranes were imaged randomly. The results were analysed by ImageJ software.

### 2.6 Colony formation assay

For the colony formation assay, 6‐well plates were coated with poly‐D‐lysine (Solarbio Life Science, China, Beijing) overnight at room temperature, and then 2000 cells were seeded. Subsequently, the culture medium was changed every three days. After culturing for 14‐21 days, the colonies were fixed with methanol (Boster Biological Technology, China,Beijing) before staining with 5% crystal violet (Solarbio Life Science, China,Beijing). Finally, the plates were photographed with a camera (Canon, Japan, Tokyo).

### 2.7 Reverse transcription‐quantitative polymerase chain reaction and western blotting

These assays were conducted as described previously.^21^ The primer sequences of reverse transcription‐quantitative polymerase chain reaction (RT‐qPCR) were as follows: METTL3_F: CAAGCTGCACTTCAGACGAA and METTL3_R: GCTTGGCGTGTGGTCTTT; PI3K_F: TCTGTCACCAATCCCAAG and PI3K_ R: TGAGCACCTCTGAAACAA; AKT_ F: CACGATACCGGCAAAGAA and AKT_R: AGGGCTGCTCAAGAAGGA; mTOR_F: TCCGAGAGATGAGTCAAGAGG and mTOR_R: CACCTTCCACTCCTATGAGGC; P70S6K_ F: TTGAGTCATCTGGGCTGT and P70S6K_ R: AAATGCTGCTTCTCGTCT; 4EBP1_ F: GGTGTTCACGAAGAGGAGGG and 4EBP1_R: ATACTGGGCAGGCGTTGG; and GAPDH_F: TGGACCTGACTTGCCGTCTA and GAPDH_ R: CCCTGTTGCTGTAGCCAAATT. The primary antibodies were as follows: METTL3 (ab195352, 1:1000; Abcam, UK, London), p‐PI3K‐p85α (Tyr607) (AP0153, 1:500; Bioworld, China, Beijing), p‐AKT (S473) (CST, USA, New York, #4058, 1:1000), p‐mTOR (Ser2448) (CST, USA, New York, #5536, 1:1000), p‐P70S6K (Ser371) (CST, USA, New York, #9208, 1:1000), p‐4EBP1 (Ser65 + Thr70) (Bioss, China, Beijing, bs‐3720R, 1:500), β‐Tubulin (Bioworld, China, Beijing, AP0064, 1:2000), and GAPDH (Bioworld, China, Beijing 1:5000). The secondary antibody was AffiniPure goat anti‐rabbit IgG (H + L) (Jackson ImmunoResearch Inc, USA, New York).

### 2.8 Subcutaneous xenograft mouse models

Male BALB/c nude mice (4‐6 weeks, 18‐20 g) were purchased from SPF (Beijing Biotechnology Co, Ltd, China, Beijing) and were fed in the Ophthalmic Animal Laboratory, Zhongshan Ophthalmic Center, Sun Yat‐sen University. To establish a subcutaneous tumour model in nude mice, 2 × 10^7^ Y79 cells (METTL3 knockdown group: shNC, shRNA1 and shRNA2; METTL3 up‐regulated group: NC and METLL3) were resuspended in 1 mL of pre‐cooled PBS, and 200 μL of the cell suspension was injected subcutaneously into the left side of the armpit to investigate tumour growth (4 × 10^6^ per mouse). Approximately 35 days later, the mice were euthanized by cervical dislocation, and the tumours were removed and weighed. All experimental procedures were conducted in line with the Guide for the Care and Use of Laboratory Animals and were approved by our institutional ethical guidelines. Tumour volume was calculated using the formula *V* = 1/2 × larger diameter × (smaller diameter)^2^.

### 2.9 Immunofluorescence staining

Tissue samples fixed with 10% formalin were imbedded in paraffin, cut into 4‐mm‐thick sections and mounted on glass slides. The sections were dewaxed in xylene and then hydrated by immersion in a gradient of ethanol solutions. Next, antigen retrieval in the sections was performed by treatment with Tris/EDTA buffer (pH 9.0, Solarbio Life Science) for 30 minutes in a microwave. After blocking with 5% goat serum (Boster Biological Technology) for 1 hour, the sections were then incubated with a primary antibody (ab195352, 1:400; METTL3, Abcam) overnight at 4°C. The sections were then washed with PBS, and Alexa Fluor 488/594‐conjugated secondary antibodies (CST) were applied for a 1 hour incubation at room temperature in the dark. Nuclei were counterstained with DAPI (Solarbio Life Science) for 15 minutes. Images were captured using a fluorescence microscope.

### 2.10 Haematoxylin and eosin staining

Subcutaneous neoplasms fixed with 10% formalin were imbedded in paraffin, cut into 5‐mm‐thick sections and mounted on glass slides. Before staining, 5‐µm‐thick tissue sections were dewaxed in xylene, rehydrated through exposure to decreasing concentrations of ethanol and washed with PBS. Then, the cells were stained with haematoxylin and eosin (H&E). After staining, sections were dehydrated by treatment with increasing concentrations of ethanol and xylene. Finally, photographs were taken with an inverted microscope (Leica, Germany, Berlin).

### 2.11 Statistical analysis

All experiments shown in the figures were conducted at least in triplicate, and IBM SPSS Statistics 21 software was used for statistical analysis. Data are presented as the average ±SD. Significant differences among the down‐regulated METTL3 groups were determined by multiple LSD’s multiple comparison test (one‐way ANOVA). In the up‐regulated groups, the significance of the difference was determined by unpaired Student's t test. Significant values are shown as *P* < 0.05 (*), *P* < 0.01 (**), *P* < 0.001 (***), *P* < 0.0001 (****) and *P* > 0.05 (#). *P* < 0.05 (*) was considered to indicate a statistically significant difference.

## 3 RESULTS

### 3.1 The expression of METTL3 in patient samples and different cell lines

**FIGURE 1:**
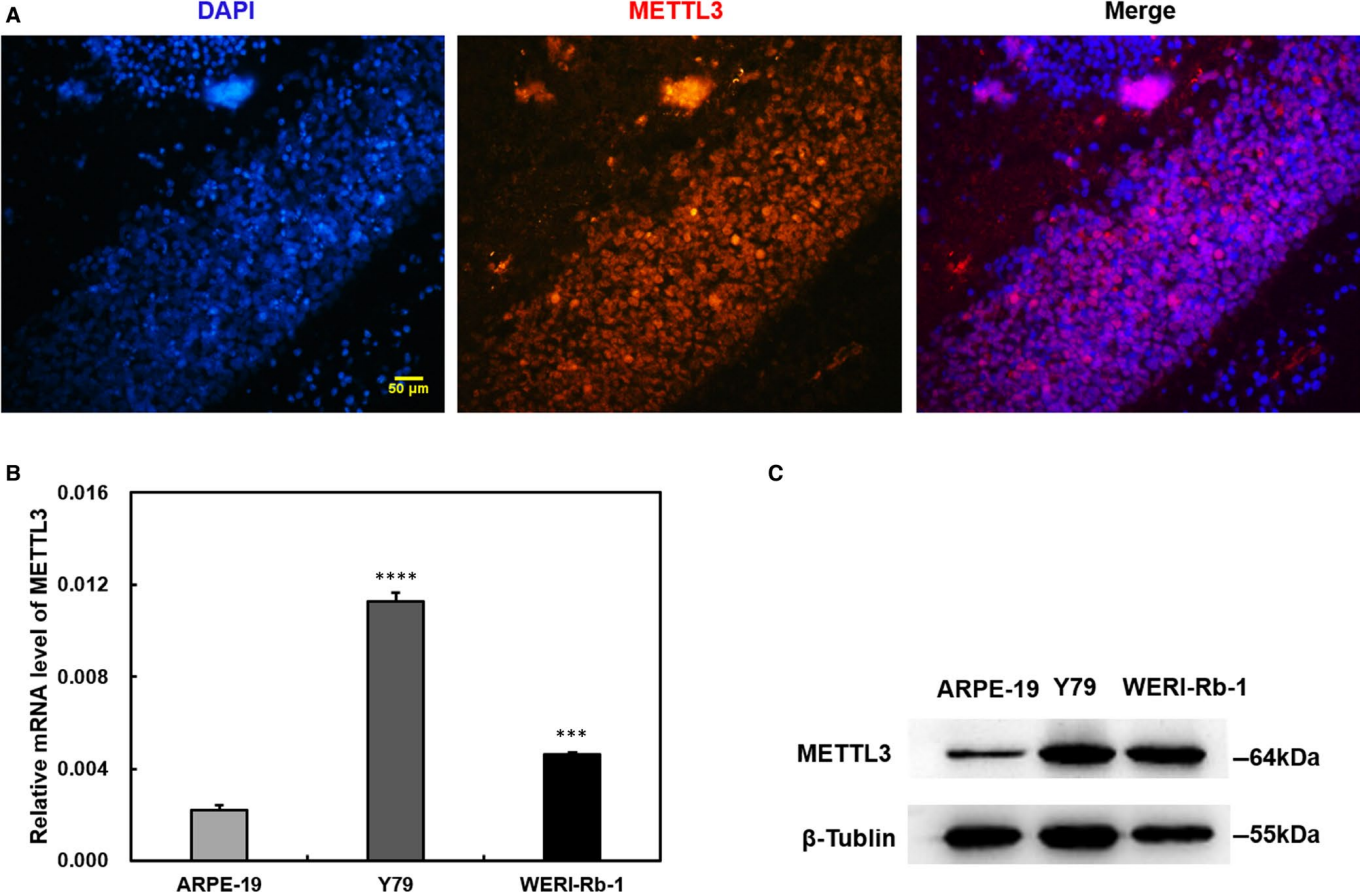
Methyltransferase‐like 3 (METTL3) expression in RB patients and retinoblastoma (RB) cells. A, Immunofluorescence assays show the expression of METTL3 in paraffin sections of tissue from RB patients. B, Compared to ARPE‐19, the mRNA levels of METTL3 in Y79 and WERI‐Rb‐1 cells was increased. C, The Western blot results were in accordance with quantitative polymerase chain reaction (B). Data are shown as the average ± SD (n = 3). *P* < 0.001(***) and*P*< 0.0001(****). The scale bars represent 50 μm

To study the function of METTL3 in the regulation of RB, we first analysed the expression of METTL3 in RB patients and found that METTL3 is expressed in RB tumour samples (Figure 1A). Since RB originates from the developing retina, we further compared the mRNA and protein levels of METTL3 in the normal ARPE‐19 and RB cell lines Y79 and WERI‐Rb‐1 by quantitative real‐time PCR and Western blot, respectively. Our results showed that the mRNA and protein levels of METTL3 in two different RB cell lines, Y79 and WERI‐Rb‐1, were higher than they were in normal ARPE‐19 cells **(**Figure 1B,C). Overall, our study revealed that METTL3 potentially regulates the progression of RB.

### 3.2 Down‐regulating METTL3 negatively regulates cell biological processes in tumour cells

**FIGURE 2:**
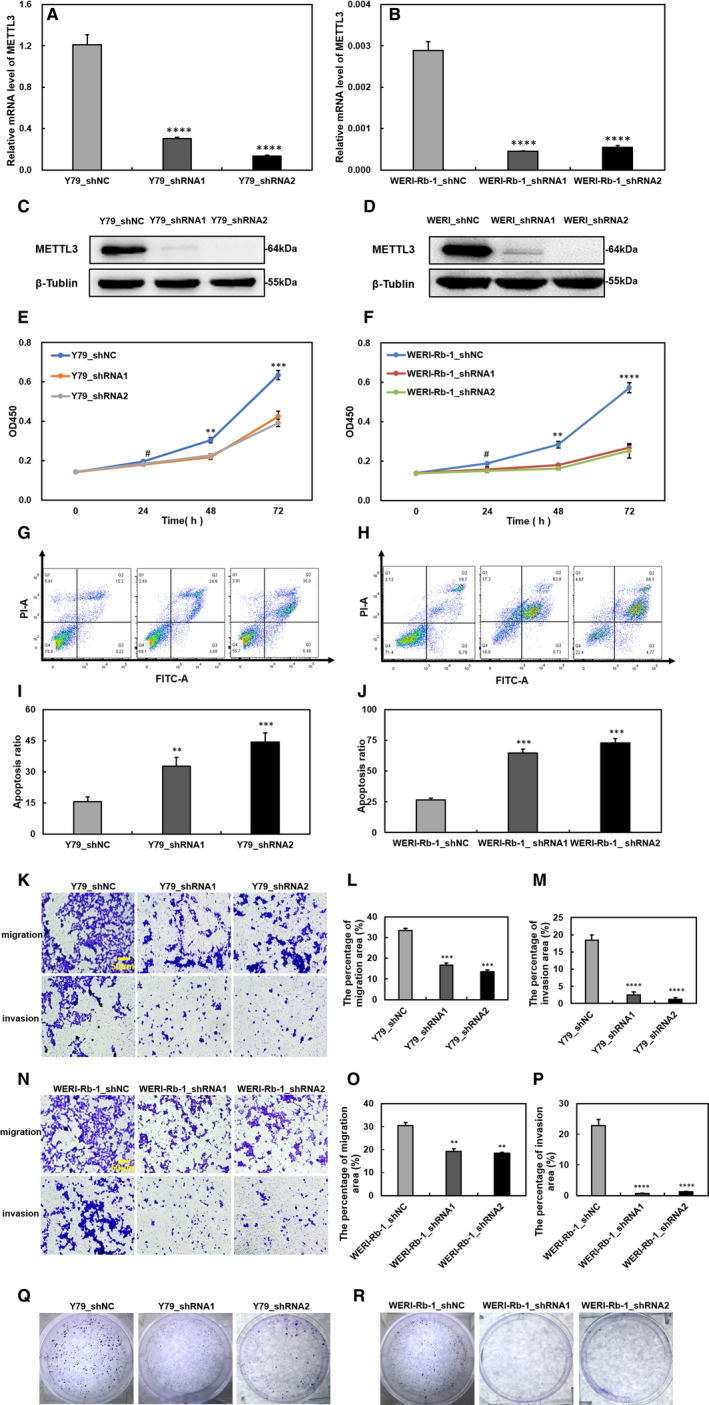
Knockdown of methyltransferase‐like 3 (METTL3) in Y79 and WERI‐Rb‐1 cells influences proliferation, apoptosis, migration, invasion and colony formation. A‐D, Confirmed the transfection efficiency of down‐regulating METTL3 at the mRNA and protein levels in Y79 (A, C) and WERI‐Rb‐1 cells (B, D). E and F, Cell viability decreased in METTL3‐down‐regulated Y79 (E) and WERI‐Rb‐1 cells (F). G and H, The apoptosis ratio increased in METTL3 knockdown Y79 (G) and WERI‐Rb‐1 cells (H). I and J, Apoptosis ratio is shown in bar chart. K and N, Cell migration and invasion were repressed by silencing METTL3 Y79 (K) and WERI‐Rb‐1 cells (N). L and M, Quantification of migratory and invasive areas in Y79 cells. O and P, Quantification of migratory and invasive areas in WERI‐Rb‐1 cells; analysis was performed with ImageJ software. Q and R, METTL3 down‐regulation inhibits cell colony formation. Data are shown as the average ± SD (n = 3). *P* <  *P* < 0.01(**), *P* < 0.001(***), *P* < 0.0001(****) and *P* > 0.05(#). The scale bars represent 50 μm

To investigate the function of METTL3 in RB, we down‐regulated METTL3 in Y79 and WERI‐Rb‐1 cells. The control group transfected with a vector (shNC) and the knockdown groups were two different shRNAs targeting diverse METTL3 sequences (shRNA1, shRNA2). The knockdown efficiency of METTL3 was verified by RT‐qPCR and Western blot (Figure 2A‐D). Then, we evaluated cell proliferation using a CCK‐8 kit and found that cell proliferation was impaired upon METTL3 depletion (Figure 2E,F). Furthermore, we showed that compared to the control levels, the apoptosis ratio increased by more than 50% in both METTL3 knockdown RB cell lines (Figure 2G‐J), suggesting that METTL3 is essential for RB cell survival. In METTL3‐down‐regulated RB cells, we found that the features of migration and invasion were obviously weakened, as measured by transwell assays (Figure 2K,N). The areas of migration and invasion were analysed by ImageJ software (Figure 2L‐P). The colony formation assay also revealed that down‐regulated METTL3 reduced the colony‐forming capacity of tumour cells (Figure 2Q,R). Taken together, our results uncovered the important roles that METTL3 plays in the regulation of RB progression in vitro.

### 3.3 Up‐regulating METTL3 positively impacted the biological processes of tumour cells

**FIGURE 3:**
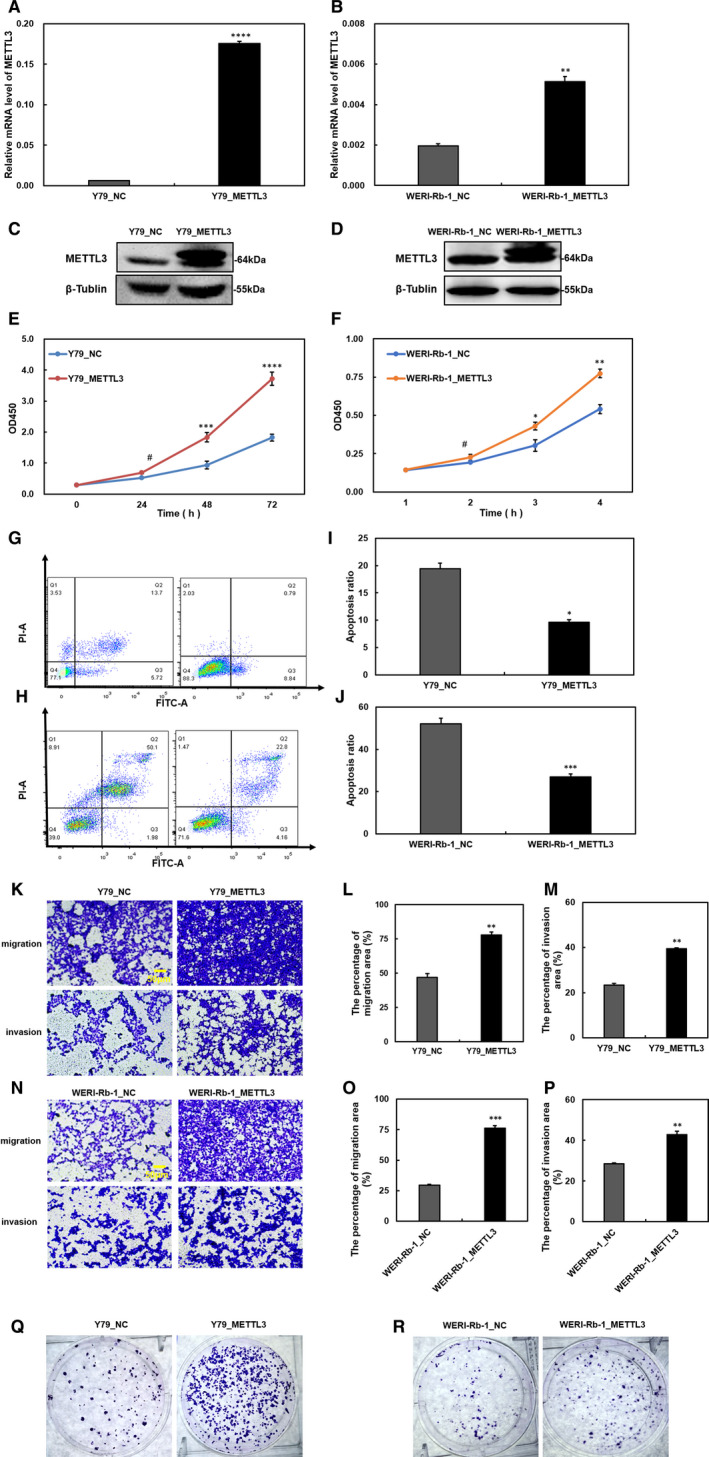
The biological features of Y79 and WERI‐Rb‐1 cells, including proliferation, apoptosis, migration, invasion and colony formation, are affected by upregulation of methyltransferase‐like 3 (METTL3). A‐D, The elevated levels of METTL3 were verified at the mRNA and protein levels in Y79 (A, C) and WERI‐Rb‐1 cells (B, D). E and F, In CCK‐8 cells, cell proliferation was enhanced by overexpressing METTL3 in Y79 (E) and WERI‐Rb‐1 cells (F). G and H, The apoptosis ratio of METTL3‐overexpressing Y79 (G) and WERI‐Rb‐1 cells (H) decreased. I and J, The apoptosis ratio data are shown in a bar chart. K and N, Cell migration and invasion are promoted by up‐regulating METTL3 in Y79 (K) and WERI‐Rb‐1 cells (N). L and M, The quantification of migratory and invasive areas in Y79. O and P, The quantification of migratory and invasive areas in WERI‐Rb‐1 cells; analysis was performed with ImageJ software. Q and R, The colony formation capacity was enhanced in METTL3‐overexpressing cells. Data are shown as the average ± SD (n = 3). *P* < 0.05(*), *P* < 0.01(**), *P* < 0.001(***), *P* < 0.0001(****) and *P* > 0.05(#). The scale bars represent 50 μm

We explored the influence of up‐regulated METTL3 in RB cells. The lentivirus constructs were used to infect Y79 and WERI‐Rb‐1 cells. The transfection efficiency of METTL3‐overexpressing cells (METTL3) and control cells (NC) was indicated RT‐qPCR and Western blot at the mRNA and protein levels (Figure 3A‐D). We found that up‐regulated METTL3 increased the proliferation of RB tumour cells (Figure 3E,F). On the other hand, the apoptosis ratio decreased by more than half in up‐regulated cells compared with NC cells (Figure 3G‐J). Transwell assays revealed that METTL3 enhances the migration and invasion of RB cells (Figure 3K‐P). Moreover, the colony‐forming capacity of tumour cells was improved in METTL3‐up‐regulated cells (Figure 3Q,R). Overall, our gain‐of‐function studies further proved the essential function of METTL3 in promoting RB progression.

### 3.4 METTL3 influences the PI3K/AKT/mTOR signalling pathway in RB cells

**FIGURE 4:**
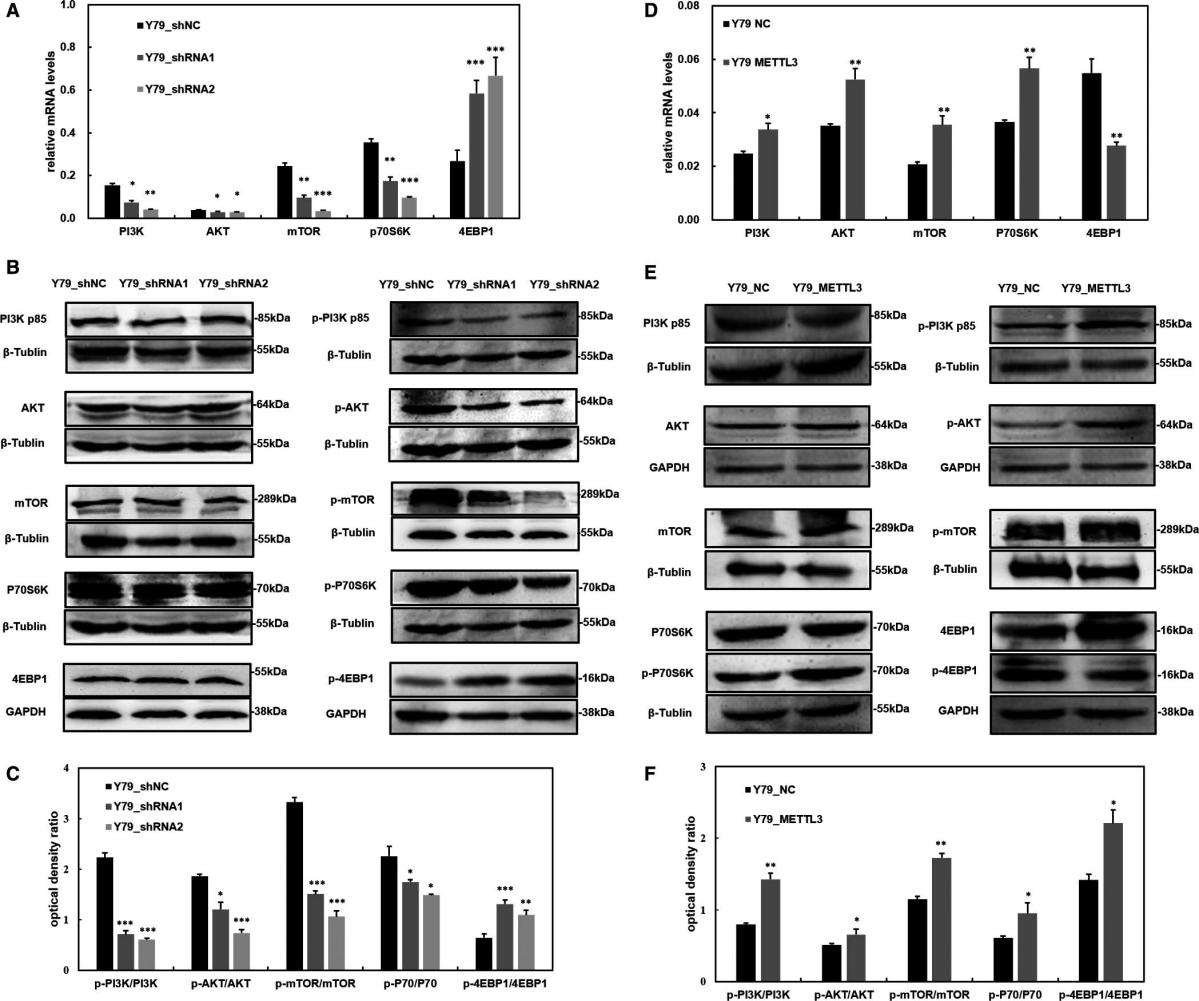
Methyltransferase‐like 3 (METTL3) influences the expression of the PI3K/AKT/mTOR pathway. A, Down‐regulated METTL3 decreases the expression of PI3K‐p85, AKT, mTOR and P70S6K but increases 4EBP1 mRNA levels. B, Western blot data show that the expression of p‐PI3K‐p85, p‐AKT, p‐mTOR and p‐P70S6K is decreased, but p‐4EBP1 is increased; non‐phosphorylated PI3K‐p85, AKT, mTOR, P70S6K and 4EBP1 have no difference. C, The optical density ratio of the Western blot. D, Up‐regulated METTL3 stimulates PI3K‐p85, AKT, mTOR and P70S6K expression at the mRNA level but reduces 4EBP1. E, The protein levels of p‐PI3K‐p85, p‐AKT, p‐mTOR and p‐P70S6K are elevated, but p‐4EBP1 is decreased; the level of non‐phosphorylated total proteins is steady. F, The optical density ratio of the Western blot. Data are shown as the average ± SD (n = 3). *P* < 0.05(*), *P* < 0.01(**), and *P* < 0.001(***)

We further studied the underlying mechanisms of METTL3 in RB regulation. Since METTL3 was reported to regulate mRNA translation in cancer cells, we decided to explore the role of METTL3 in the regulation of the PI3K/AKT/mTOR signalling pathway. We found that METTL3 knockdown decreased the mRNA levels of PI3K‐p85, AKT, mTOR and P70S6K, but it increased 4EBP1 mRNA expression (Figure 4A). In addition, Western blot analysis revealed that there was no significant difference in the levels of non‐phosphorylated PI3K‐p85, AKT, mTOR and P70S6K and 4EBP1. Moreover, while phosphorylated PI3K‐p85, AKT, mTOR and P70S6K decreased, p‐4EBP1 increased in METTL3‐down‐regulated cells (Figure 4B,C). This indicated that the activity of this pathway was decreased in METTL3‐depleted RB cells. On the other hand, we found that the mRNA levels of PI3K‐p85, AKT, mTOR and P70S6K increased in METTL3‐up‐regulated cells, but 4EBP1 decreased (Figure 4D). METTL3 overexpression in RB cells increased p‐PI3K‐p85, p‐AKT, p‐mTOR and p‐P70S6K levels, but it decreased p‐4EBP1, while the levels of non‐phosphorylated PI3K‐p85, AKT, mTOR and P70S6K and 4EBP1 were not significantly different (Figure 4E,F). These results demonstrated that METTL3 has an impact on the PI3K‐AKT‐mTOR‐P70S6K/4EBP1 pathway.

### 3.5 METTL3 regulates cell proliferation, migration and invasion through the PI3K/AKT/mTOR signalling pathway

**FIGURE 5:**
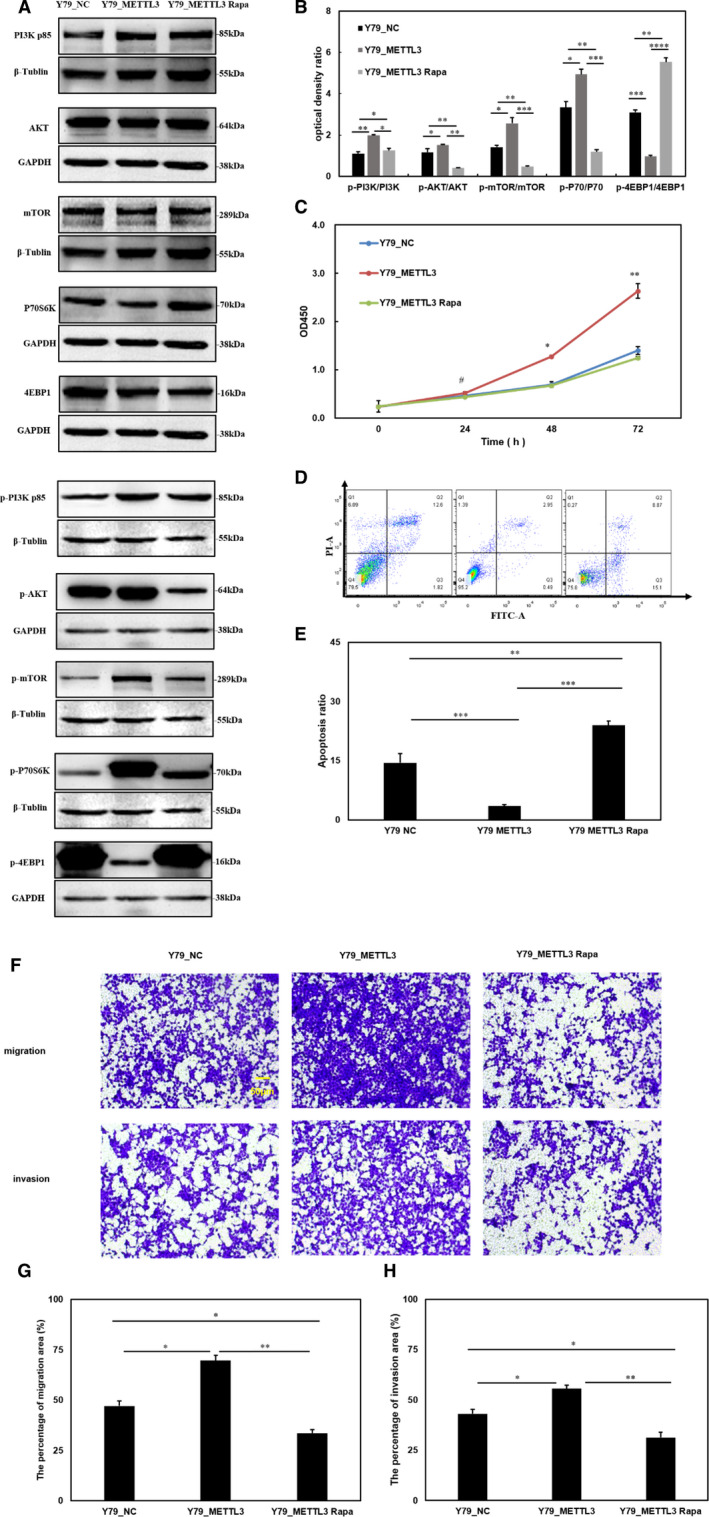
Methyltransferase‐like 3 (METTL3) regulates cell proliferation, apoptosis, migration and invasion via the PI3K/AKT/mTOR pathway. A, Rapamycin inhibits the expression of p‐PI3K‐p85, p‐AKT, p‐mTOR and p‐P70S6K but elevates the expression of p‐4EBP1. The total protein levels of PI3K‐p85, AKT, mTOR, P70S6K and 4EBP1 were not different. B, The statistical histogram of the phosphorylated proteins/total proteins. C, The cell proliferation results show that the stimulatory function of METTL3 is lost after rapamycin treatment. D and E, The apoptosis ratio changes were higher in the METTL3 Rapa group than in the METTL3 group and NC group. F, The cell migration and invasion abilities in the METTL3 Rapa groups were weaker than those in the METTL3 groups and had no statistical difference with NC groups. G and H, Quantification of migratory and invasive areas was analysed by ImageJ software. Data are shown as the average ± SD (n = 3). *P* < 0.05(*), *P* < 0.01(**), *P* < 0.001(***), *P* < 0.0001(****) and *P* > 0.05(#). The scale bars represent 50 μm

We used 10 μmol/L rapamycin, an inhibitor of mTOR, to restrict PI3K/AKT/mTOR signalling in up‐regulated METTL3 cells and then tested the changes in biological processes in cells. First, we examined the inhibitory effect of rapamycin on the PI3K/AKT/mTOR signalling pathway (Figure 5A,B). Compared with the METTL3 group, the cell proliferation in the METTL3 Rapa group decreased, and there was nearly no difference from the NC group (Figure 5C). The apoptosis ratio was increased in the METTL3 Rapa group compared with that of the METTL3 group and NC group (Figure 5D,E). Cell migration and invasion were also repressed after rapamycin treatment (Figure 5F‐H). We found that in up‐regulated METTL3 cells, the positive effects of RB cells were eliminated by inhibiting the PI3K/AKT/mTOR signalling pathway using rapamycin. These results indicate that METTL3 regulates the proliferation, apoptosis, migration and invasion of RB cells through the PI3K/AKT/mTOR signalling pathway.

### 3.6 METTL3 promotes the growth of RB cells in vivo

**FIGURE 6:**
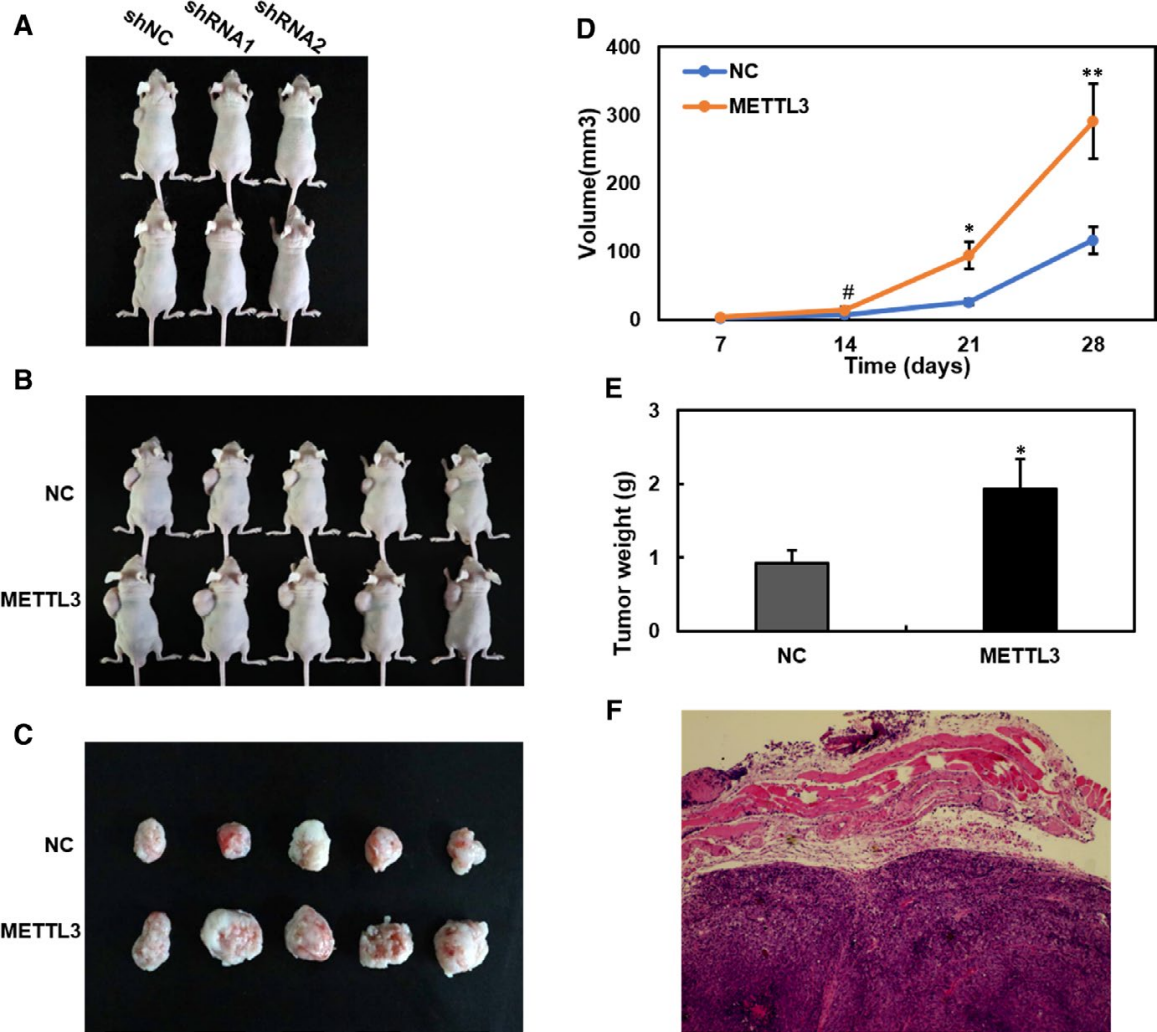
Methyltransferase‐like 3 (METTL3) promotes tumorigenicity in vivo. A, Down‐regulated METTL3 obviously restrains subcutaneous tumorigenesis. B, Up‐regulated METTL3 facilitates tumour growth. C, The neoplasm is removed from the subcutaneous tissue. D, The volume growth curve shows that METTL3 facilitated the growth process of tumours. E, The weight difference of tumours is shown in the histogram. F, Haematoxylin and eosin staining of tumours. Data are shown as the average ± SD (n = 5). *P* < 0.05(*), *P* < 0.01(**), and *P* > 0.05(#)

To determine whether METTL3 affects the growth of RB cells in vivo, we established a subcutaneous tumour model by injecting METTL3‐down‐regulated Y79 cells and control cells (shNC, shRNA1 and shRNA2) into nude mice. Compared with the control (shNC), the tumours in the METTL3 knockdown groups (shRNA1 and shRNA2) did not grow or were gradually absorbed under the skin (Figure 6A). Next, we further investigated the function of METTL3 overexpression in RB tumorigenesis in the subcutaneous tumour model. Our results showed that METTL3 overexpression significantly increased the volume (Figure 6B‐D) and weight (Figure 6E) of RB tumours in vivo. The H&E staining results showed the histological characteristics of RB tumours (Figure 6F). In summary, these results demonstrated that METTL3 plays important functions in promoting the growth of RB in vivo.

## 4 DISCUSSION

In this manuscript, we first revealed that METTL3 is a critical factor promoting RB progression. Our results revealed that METTL3 knockdown decreases RB cell proliferation, migration, invasion and tumorigenesis, while overexpression of METTL3 promotes RB progression in vitro and in vivo. Because METTL3 down‐regulation had a great impact on the vitality of RB cells in vitro, in our xenograft model, the METTL3 knockdown group did not form stable hypodermal neoplasms in vivo. The in vitro and in vivo results showed that METTL3 is a vital factor in RB and may be a potential therapeutic target for RB therapy.

Studies have shown that RB is prone to invade adjacent tissues.^4^, ^5^ Therefore, preventing metastasis is of great significance for controlling RB. We found that down‐regulating METTL3 could inhibit the migration and invasion of RB cells, and up‐regulating METTL3 enhanced these characteristics. Thus, METTL3 may be a novel target for RB treatment. Chen et al^22^ reported that the functional expression of METTL3 could contribute to the progression of hepatocellular cancer development. In their studies, the tumour suppressor SOCS2 was identified to be the direct downstream target of METTL3. It was shown that dysregulation of METTL3 silenced the function of the tumour suppressor SOCS2 and promoted liver carcinogenesis. Previous studies indicated the role of METTL3 in the self‐renewal and tumorigenesis of glioblastoma stem cells.^23^ Retinoblastoma originates from the developing retina, which is part of the nervous system. This research provides a reliable basis for our exploration. Recently, some studies on nanoparticles have clarified that nano‐based therapy could inhibit organ damage in some relapsed and refractory malignancies.^24^, ^25^ Our study provided new insights into the role of METTL3 in the regulation of cancer progression.

Methyltransferase‐like 3 has multiple functions by mediating different effector molecules or pathways. Our results revealed that METTL3 regulates the PI3K‐AKT‐mTOR‐P70S6K/4EBP1 pathway in Y79 cells. The PI3K/AKT/mTOR pathway is a key signalling cascade that participates in numerous physiological and pathological conditions. The PI3K/AKT/mTOR pathway regulates the translation of mRNAs that encode pro‐oncogenic proteins, leading to malignant cell survival in various cancers.^26^ This pathway is pivotal in modulating cancer proliferation, migration and invasion by altering some genes that could impact biological processes in tumour cells.^27^, ^28^ Members of the PI3K/AKT/mTOR pathway were inactivated in METTL3‐down‐regulated cells but activated in METTL3‐up‐regulated cells. After treatment with an mTOR inhibitor, the PI3K/AKT/mTOR pathway was inactivated. At the same time, the function of METTL3, which can promote cell biological processes, disappeared. Our data suggested that METTL3 mediated the biological features of RB cells through the PI3K/AKT/mTOR/P70S6K/4EBP1 pathway. Therefore, we think that the METTL3/PI3K/AKT/mTOR signalling axis may be an efficient target for the treatment of RB.

In summary, we revealed that METTL3 could regulate oncogenesis in RB, suggesting that METTL3 is an oncogene in RB. Our findings uncovered novel insights into the function and mechanism of METTL3 in promoting RB progression.

## CONFLICT OF INTEREST

No potential conflicts of interest were disclosed.

## AUTHOR CONTRIBUTION

**Han Zhang:** Data curation (equal); Formal analysis (equal); Investigation (lead); Methodology (equal); Writing‐original draft (lead). **Ping Zhang:** Methodology (equal); Resources (lead); Writing‐review & editing (equal). **Chongde Long:** Conceptualization (equal); Methodology (equal); Writing‐review & editing (equal). **Xinqi Ma:** Formal analysis (equal); Investigation (equal); Methodology (equal); Software (equal); Validation (equal); Writing‐review & editing (equal). **Hao Huang:** Conceptualization (equal); Software (equal); Writing‐review & editing (equal). **Xielan Kuang:** Investigation (equal); Methodology (equal); Software (equal). **Han Du:** Conceptualization (equal); Data curation (equal); Methodology (equal). **Han Tang:** Methodology (equal); Software (equal). **Xiangtian Ling:** Conceptualization (equal); Formal analysis (equal); Methodology (equal). **Jie Ning:** Methodology (lead); Resources (equal); Software (equal). **Huijun Liu:** Methodology (equal); Software (equal). **Xizhi Deng:** Conceptualization (equal); Formal analysis (equal); Methodology (equal). **Yuxiu Zou:** Conceptualization (equal); Data curation (equal); Software (equal). **Renchun Wang:** Methodology (equal); Software (equal). **Hao Cheng:** Writing‐review & editing (equal). **Shuibin Lin:** Methodology (equal); Supervision (equal); Writing‐review & editing (equal). **Qingjiong Zhang:** Supervision (equal); Writing‐review & editing (equal). **Jianhua Yan:** Conceptualization (equal); Resources (equal); Supervision (equal). **Huangxuan Shen:** Conceptualization (equal); Project administration (equal); Supervision (lead); Validation (lead); Writing‐review & editing (equal).

## Data Availability

All data included in this study are available upon reasonable request by contact with the corresponding author.
